# Surgically Relative Risk Factors for Lower Colorectal Anastomotic Dehiscence and Rectovaginal Fistulas in Complex Deep Endometriosis Cases: A Single-Center Retrospective–Prospective Cohort Study

**DOI:** 10.3390/jcm15072630

**Published:** 2026-03-30

**Authors:** Krzysztof Nowak, Alicja Dąbrowska, Maja Mrugała, Ewa Milnerowicz-Nabzdyk

**Affiliations:** Oncological Gynecology Department and Reference Endometriosis Center, Center of Oncology in Opole, Faculty of Medicine, Opole University, 45-061 Opole, Poland; knowakmd@gmail.com (K.N.);

**Keywords:** bowel endometriosis, laparoscopy, complication, prevention

## Abstract

**Background**: Bowel surgery is a key component of advanced deep endometriosis management, with anastomotic leakage representing the most serious postoperative complication. This study aimed to identify risk factors for dehiscence after lower colorectal anastomosis and to determine effective preventive measures. **Methods**: This retrospective/prospective study included 425 consecutive patients aged 37.7 ± 6.0 years with laparoscopical bowel resection due to multiorgan complex deep endometriosis. All bowel surgeries were performed with use of indocyanine green (ICG). Many technical aspects of surgery and preventive procedures were analyzed which could impact leakage risk of surgery. **Results**: Endometriotic nodules were resected with segmental bowel resection (*n* = 294; 69.8%), discoid bowel resection (*n* = 84; 20.0%), and shaving procedure (*n* = 43; 10.2%). A total of 12 dehiscence events occurred (2.8%), including intraperitoneal leakage (*n* = 1; 0.2%), rectovaginal fistula (RVF) (*n* = 10; 2.3%), and rectoureteral fistula (*n* = 1; 0.2%); no rectovesical fistulas were observed. RVF developed only following segmental resections. Protective measures used during lower bowel procedures included fibrin glue (*n* = 375; 88.2%), omental flaps (*n* = 86; 20.2%), reinforcing sutures (*n* = 33; 7.8%), protective stomas (*n* = 25; 5.9%), and ghost stomas (*n* = 14; 3.3%). Among patients who developed RVFs, 90% had no protective stoma, and these cases were predominantly associated with low (from 6 to <8 cm; *n* = 4/77; 5.2%) and very low (from 5 to <6 cm; *n* = 4/10; 40%) anastomoses. In very low anastomoses (*n* = 4), 1 RVF occurred despite a protective stoma but there existed other strong risk factors, such as levator ani infiltration and vagina opening, whereas 3 others RVF developed in patients without a protective stoma. Notably, in ultra-low anastomoses (<5 cm), protective stomas prevented the anastomosis in 100%, and no fistula was observed (*n* = 3). The following factors were associated with the increased rate of RVF: segmental resection (*p* = 0.0355), low and very low anastomosis (*p* = 0.0010), lateral infiltration of the levator (*p* < 0.0001), concomitant hysterectomy or vaginal opening (*p* = 0.051), and prolonged operative time (*p* = 0.0010), *Clostridioides difficile* infection (*p* = 0.0001). **Conclusions:** RVFs occurred mainly after segmental resection (no other type of bowel resection), with very low anastomosis (5–6 cm from anal verge), in patients with levator ani infiltration and concomitant vaginal or uterine surgery; in such situations, discoid resection is the safer option. Despite the complexity of procedures, preventive strategies maintained a low overall RVF rate; no RVFs occurred in ultra low anastomoses (<5), indicating effective prevention with protective stomas.

## 1. Introduction

The bowel is the most common extragenital deep endometriosis location. Bowel surgery constitutes a major component of operative management in advanced deep endometriosis, affecting between 5% and 12% of patients with deep endometriosis [1]. Individualized, tailored procedures are critical to achieve optimal outcomes and minimize complications [2]. Surgical planning must balance complete excision with the prevention of intraoperative and postoperative complications, while preserving bowel and bladder function and, when relevant, fertility. This multifactorial decision requires robust surgical judgment grounded in detailed knowledge of pelvic anatomy and endometriosis. It also varies with the surgeon’s experience and technical expertise [3,4]. In many clinical scenarios, bowel nodules represent only one manifestation of a multi-compartment, multi-organ disease, requiring a comprehensive, multidisciplinary surgical approach [5,6]. Therefore, prudent preventive management is essential to ensure surgical success and optimize patient outcomes.

Among postoperative adverse events, bowel complications, particularly anastomotic leakage, are associated with the greatest morbidity and can lead to life-threatening conditions [7]. For this reason, preventive management is essential and should be implemented across the entire perioperative pathway. This includes preoperative optimization and general measures, intraoperative preventive techniques, and postoperative monitoring using clinical assessment, biochemical tests, and imaging when indicated [7]. Preventive measures are crucial to reduce the risk of anastomotic leakage and its clinical consequences. Such strategies have been reported by other researchers [7,8,9,10]. However, large studies including patients with advanced endometriosis and complex procedures are still needed. This evidence is essential to optimize management in those at the highest risk. Treatment should be individualized to align with each woman’s preferences and the specific characteristics and extent of the disease [4]. Perfect planning and comprehensive preoperative assessment are essential to reduce complication rates. Several systems have been proposed to provide an objective assessment of predictive complexity of surgery; among them, the Enzian classification warrants particular attention. Accurate preoperative staging may help anticipate the need for multi-organ surgery and stratify the associated operative risk. Proper estimation can help to predict the level of risk connected with potential multiorgan surgery [4,11,12,13].

This study focuses on identifying the most important determinants of rectosigmoid anastomotic dehiscence. Rectosigmoid anastomosis is the most common anastomosis performed in bowel endometriosis surgery. This reflects the predominant distribution of intestinal disease, most frequently involving the rectum (≈90%) and the sigmoid colon (≈50%) [14]. Other bowel sites may also be affected, including the appendix, cecum, and terminal ileum. Anastomoses in these segments pose distinct clinical challenges and will be addressed in a separate publication. However, the impact of upper bowel procedures on the risk of lower colorectal anastomotic leakage was included as one of the study objectives. The aim of this study was to evaluate risk factors for anastomotic leakage after lower colorectal anastomosis and to identify effective preventive measures to reduce the risk of this complication.

## 2. Materials and Methods

### 2.1. Study Design

This retrospective–prospective cohort study enrolled 425 consecutive patients with bowel resection for deep endometriosis who underwent surgery at the Opole Oncology Center (OCO), Opole, Poland, from 10 January 2020 to 31 December 2025. Data from 10 January 2020 to 11 January 2023 were collected retrospectively, whereas data from 12 January 2023 to 31 December 2025 were collected prospectively. The OCO serves as a regional gynecologic oncology reference center and a referral center for advanced deep endometriosis. The study protocol received approval from the Ethics Committee at the University of Opole (approval no: UO/0001/KB/2023).

### 2.2. Statistical Analysis

Data were collected and stored in a Microsoft Excel file (Microsoft Excel 2013; Microsoft Corp., Redmond, WA, USA) for preprocessing. Statistical analyses were conducted with MedCalc, version 19.5.3 (MedCalc Software Ltd., Ostend, Belgium). Continuous variables were reported as mean ± standard deviation (SD), and categorical variables as counts and percentages. Normality was evaluated using the Shapiro–Wilk test. Between-group differences were assessed with the Mann–Whitney U test (two groups) or the Kruskal–Wallis test (≥3 groups). Categorical data were compared using the Chi-square test. Statistical significance was set at *p* < 0.05.

### 2.3. Eligibility Criteria

Eligible participants were all patients who underwent bowel resection for deep endometriosis at OCO between 2020 and 2025. Specifically, this included symptomatic patients with bowel endometriosis identified on ultrasound or MRI and subsequently confirmed by postoperative histopathological examination. Patients were also included when a bowel lesion was detected incidentally during surgery for endometriosis in other organs, excised, and confirmed as endometriosis on histopathology. Exclusion criteria comprised the presence of any infectious disease, including COVID-19, as well as cases in which histopathological examination did not confirm bowel endometriosis. Regarding the excluded COVID-19 case: one patient developed an acute COVID-19 infection on the first postoperative day (the admission test was negative) and subsequently developed fulminant hemorrhagic enteritis requiring completely different postoperative management compared with the remaining patients. Because this situation represented a unique clinical course unrelated to the surgical factors analyzed in our study, the case was excluded to avoid introducing bias.

In total, 425 patients with bowel endometriosis confirmed by final histopathology met eligibility criteria and were included in the study. The mean age of patients was 37.7 ± 6.0 years, and the mean body mass index (BMI) was 22.2 ± 3.2 kg/m^2^. The mean number of previous surgeries was 2.3 ± 1.3 among patients with at least one previous surgery, but there were patients with as many as 10 prior procedures done before.

### 2.4. Examinations Used to Verify Eligibility Criteria

Preoperative imaging assessment included transvaginal ultrasound (TVUS) and pelvic magnetic resonance imaging (MRI). TVUS, performed by the first surgeon E.M.-N., was used as the first-line modality to detect rectosigmoid nodules, assess lesion size and depth of infiltration, and evaluate the relationship between bowel lesions and adjacent pelvic structures. A pelvic MRI was performed to provide comprehensive mapping of disease extent, particularly in cases of suspected multicompartment involvement. MRI allowed detailed assessment of rectal wall infiltration, measurement of the distance from the anal verge, and evaluation of associated lesions in other pelvic compartments, thereby supporting preoperative planning. MRI scans were reviewed by the first surgeon (E.M.-N.), together with a detailed medical history and bimanual examination per vaginam and per rectum. Based on this comprehensive assessment, patients were qualified for the planned procedure by the first surgeon (E.M.-N.).

### 2.5. Characteristics of Lower Bowel Anastomoses

All cases of bowel endometriosis were managed laparoscopically with no conversions to laparotomy. Intraoperative visualization was achieved using the RUBINA^®^ imaging system with a 0-degree laparoscope (KARL STORZ SE & Co. KG, Tuttlingen, Germany). Linear and circular staplers (Medtronic, Minneapolis, MN, USA) were used for anastomosis and bowel transection. SurgiSleeve (Medtronic, Minneapolis, MN, USA) was used to protect incision sites during surgery.

Circular staplers were used in 363 procedures. They were applied for lower colorectal anastomoses, such as end-to-end, side-to-end, or end-to-side (*n* = 279), and for discoid resections (*n* = 84). Table 1 presents the distribution of circular stapler sizes used. Linear staplers were used in 62 procedures for side-to-side anastomoses and for transection of the proximal and distal bowel ends. Among the 62 procedures with side-to-side anastomosis, 15 were performed on the sigmoid colon, while the remaining cases involved the upper bowel, including the ascending colon, cecum, and ileum.

Circular staplers were used for colorectal anastomoses of the lower large bowel, including end-to-end, side-to-end, and end-to-side configurations, as well as for discoid resections. All circular staplers were equipped with either double- or triple-staggered rows of staples. Linear staplers were used for side-to-side anastomoses and for transection of the proximal and distal bowel ends. Stapler cartridges of 60 mm or 45 mm were applied, depending on the surgical indication. All side-to-side anastomoses were performed using a 60 mm linear stapler. All linear staplers were equipped with triple-staggered rows of staples.

Bowel endometriosis was managed using the following surgical techniques, selected according to lesion size, depth of infiltration, and degree of bowel stenosis. Shaving resection is a procedure consisting of excision of the lesion from the wall of the large bowel without full-thickness resection, reserved for superficial and small nodules. Discoid resection is a procedure involving full-thickness excision of the bowel lesion using a circular stapler introduced transanally. It is reserved for lesions up to 3 cm in length, infiltrating no deeper than 7 mm into the bowel wall, without luminal stenosis preventing insertion of the stapler above the lesion. This technique is feasible for lesions located on the anterior bowel wall, but not too high. The limitation is the accessibility of the changes for transanal insertion of the circular stapler. Segmental resection is a procedure consisting of the removal of a bowel segment containing the lesion. It is indicated for nodules larger than 3 cm, full-thickness lesions exceeding 7 mm in depth, or in cases of bowel lumen stenosis greater than 50%.

The cohort differed with respect to the surgical procedures performed. All resected bowel segments are summarized in Table 2, and the types of lower colorectal procedures (rectum, sigmoid colon, and combined rectum with sigmoid colon) are presented in Table 3.

Discoid anastomoses were classified according to the number of firings of the circular stapler. A **single discoid resection** involved one full-thickness excision of the one-time anterior bowel wall performed with a single firing of the circular stapler. A **double discoid resection** involved two sequential excisions: after the initial circular stapler firing, a second firing was performed to remove residual endometriotic tissue by encompassing the anastomotic line together with the surrounding infiltrate. This approach was typically used for flat but more extensive lesions (>3 cm) or for nodules thicker than 7 mm. A **triple discoid resection** followed the same principle but required three circular stapler firings to achieve complete excision. This technique was most often applied in large and low-located lesions, in which segmental resection was considered to carry an unacceptably high risk of anastomotic leakage.

Bowel anastomoses were categorized according to the anastomotic configuration and stapling technique. Side-to-side (ss) anastomosis was performed by joining the walls of two bowel segments using a linear stapler. Side-to-end (se) anastomosis involved connecting the lateral wall of the distal segment to the end of the proximal segment using a circular stapler. End-to-side (es) anastomosis was created by connecting the end of the distal segment to the lateral wall of the proximal segment using a circular stapler. End-to-end (ee) anastomosis consisted of joining the ends of the two bowel segments using a circular stapler. Details are shown in Table 4.

In lower segmental resections, the method of placement of the circular stapler anvil (“anvil placement”) was categorized into three approaches: *per vaginam*, *per rectum*, and *per* Surgisleeve. In the *per vaginam* approach, the proximal bowel segment was exteriorized through the vagina and the anvil was introduced into the colon via this route; in the present study, a modified variant of this technique (fishing *per vaginam*; FP) was used. In the per *rectum approach*, the anvil could be introduced transanally into the colon either after exteriorization of the proximal segment through the anus or without exteriorization using a fishing technique. In our group of patients, we used only the fishing technique. In the per Surgisleeve approach (SS), the proximal bowel segment was exteriorized through the abdominal wall, and the anvil was introduced via the Surgisleeve by extending the skin left side incision for the troacar to 3 cm in length.

Operational variants were defined as follows. Fishing (F) refers to transanal introduction of the anvil, secured with the long thread, into the proximal part of bowel, followed by bowel transection above the nodule, intra-abdominal exteriorization (“popping out”) of the anvil, transection the distal stump of bowel below the nodule, removal the segment of bowel with the nodule out of abdomen and intracorporeal anastomosis both ends of the bowel.

Fishing *per vaginam* (FP) is our modified version of the standard vaginal approach, characterized by an alternative method of anvil placement and reduced bowel mobilization. In this technique, the anvil is placed through a bowel segment exteriorized via the vagina. The bowel wall is incised transversely at the level of the lesion to a width that allows insertion of the anvil. The opening is then sutured, and the bowel stump is returned to the abdominal cavity. Next, the bowel is transected transversely above the lesion and above the suture, and the anvil is exteriorized intra-abdominally, and an end-to-end, end-to-side, or side-to-end anastomosis is done. The specimens are evacuated through the vagina. This technique preserves the length of mobilized bowel, resulting in a shorter resection segment and a more bowel-sparing approach for the patient. The bowel transection is perfectly guided intra-abdominally using indocyanine green (ICG) mapping of bowel vascularity. Surgisleeve (SS) refers to anvil introduction through the abdominal wall using a Surgisleeve device. Mostly, the bowel with the nodule is transected with the use of a purse–string clamp and cold scalpel, then an anvil is inserted, and an intra-abdominal anastomosis end-to-end or side-to-end is done. Details are shown in Table 5.

### 2.6. Patient Preparation for Surgery

All patients scheduled for bowel resection underwent prehabilitation, preceded by a dedicated prehabilitation consultation conducted either in person or online. The consultation involved the patient and a multidisciplinary team, including a gynecologic surgeon from the operative team, a dietitian, a psychologist, a physiotherapist, and a stoma care nurse; in selected cases, an anesthesiologist also participated.

During the visit, a comprehensive medical history was obtained. Based on the patient’s comorbidities, nutritional status, physical condition, psychological well-being, and the planned surgical procedure, an individualized preparation program was developed. This program included a tailored high-protein diet, a structured set of physical and breathing exercises, and medical recommendations regarding potential modification of ongoing therapy when required due to existing health conditions.

Prehabilitation consultations were conducted at least two weeks before the planned hospital admission for surgery.

### 2.7. Bowel Preparation and Perioperative Diet

Patients followed a high-protein diet for two weeks before surgery, with caloric intake adjusted individually. They were advised to consume approximately 3 L of water daily during this period. Two days before surgery, a high-protein liquid diet was introduced. On the day preceding the procedure, a bowel Enema was administered and repeated on the morning of surgery. During the first two postoperative days, patients remained on a high-protein liquid diet. However, if bowel movements occurred earlier, for example, on the first postoperative day, a light diet was introduced immediately. If no stool was passed, the diet was advanced to a light diet by postoperative day three at the latest.

### 2.8. Perioperative Antibiotic Prophylaxis

Two hours before surgery, patients received intravenous metronidazole. After completion of the surgery, a third-generation cephalosporin was administered. Given that the majority of operations involved multiorgan surgery, antibiotic prophylaxis was continued for 48–72 h postoperatively. Probiotic supplementation was initiated concurrently and was recommended to be continued for several months after hospital discharge.

### 2.9. Thromboprophylaxis

Patients received perioperative thromboprophylaxis starting 12 h before surgery with low-molecular-weight heparin administered at a prophylactic dose, unless therapeutic dosing was indicated. Anticoagulation was continued for up to 14 days postoperatively. Graduated compression stockings with an individually selected level of compression were also used.

### 2.10. Peritoneal Drainage and Bladder Catheterization

A peritoneal drain was placed intraoperatively in all patients. In the majority of cases, the drain was removed after patient mobilization, typically within 12–24 h after surgery. Urinary catheterization was performed in the operating room. In the absence of bladder opening or very low colorectal resection, the catheter was removed together with the drain after patient mobilization.

### 2.11. Surgery Involving the Urinary Tract

Among 421 procedures involving lower bowel resection, in 63 cases (15.0%), lower bowel resections were performed without any concomitant uterine or vaginal procedures, while 358 cases (85%) had the following concomitant surgery on the uterus and/or vagina included:

- Hysterectomy without additional vaginal surgery (*n* = 174; 41.3%);
- Hysterectomy with vaginal shaving (*n* = 96; 22.8%);
- Vaginal shaving without hysterectomy (*n* = 64; 15.2%);
- Excision of vaginal infiltrate with vaginal opening without hysterectomy (*n* = 19; 4.5%);
- Trachelectomy (*n* = 5; 1.2%).

Such a high rate of hysterectomy accompanying bowel resections resulted from the fact that the predominant group of patients consisted of women after multiple previous surgeries, with multi-organ endometriosis, and who had completed their reproductive plans (the average age was 37.65 ± 5.97 with a range between 24 and 57 years).

In the study group, bowel resections were performed without concomitant bladder surgery in 344 cases (81.7%). The remaining patients underwent concomitant bladder procedures as follows:

- Full-thickness bladder resection with bladder opening (*n* = 25; 5.9%);
- Non-full-thickness resection without bladder opening using mucosal skinning technique (*n* = 14; 3.3%);
- Non-full-thickness resection without bladder opening using shaving technique (*n* = 38; 9.1%).

Types of ureteral procedures accompanying bowel resections are presented in Table 6.

### 2.12. Protective Measures for Colorectal Anastomoses

During surgery, protective measures for colorectal anastomoses were applied, including fibrin glue (*n* = 352; 93%), omental flaps (*n* = 82; 21.7%), reinforcing sutures (*n* = 26; 6.9%), protective stomas (*n* = 25; 6.6%), and Ghost stomas (*n* = 14; 3.7%). Fibrin glue was used in all patients from the date it was incorporated into the perioperative protocol. An omental flap was used when the bowel anastomosis was in close proximity to vaginal and/or bladder suturing, provided that the omentum was sufficient to create a tension-free flap. Reinforcing sutures were applied when the bowel wall at the anastomotic site was thin.

In total, 39 stomas were created. This included 25 (51.0%) protective stomas and 14 (28.6%) Ghost stomas.

Indications for the creation of a protective stoma were as follows:

- Low (from 6 to <8 cm), very low (from 5 to <6 cm), or ultra-low (<5 cm) colorectal anastomosis with concomitant vaginal opening and/or resection of a lesion involving the levator ani muscle;
- Low, very low, or ultra-low anastomosis with a thin bowel wall and concomitant ureteral procedures and vaginal shaving;
- Multisegment bowel resections or multiorgan resections with a low/very low/ultra-low anastomosis, with or without vaginal opening;
- Multisegment bowel resections in patients with bronchial asthma treated with systemic steroids;
- Multisegment bowel resection complicated by a stapler-related technical error (incomplete staple line), managed with re-resection and protective stoma formation.

Indications for Ghost stoma protection were as follows:

- Impaired vascularization of the bowel segment combined with multiorgan surgery;
- Extensive adhesions and substantial small bowel dissection requiring multiple sutures, particularly in patients with a history of tubo-ovarian abscess;
- Multisegment bowel resection;
- Low anastomosis with concomitant vaginal opening;
- Multisegment bowel resection with ureteral dissection and vaginal opening;
- Multiple anastomoses with vaginal opening;
- Suspected microleakage or a history of perioperative pelvic inflammatory processes.

### 2.13. Details on Anastomotic Complications

Lower colorectal anastomotic leakage was categorized as intraperitoneal leakage or fistula formation. In 425 patients, 12 leakages were identified (2.8%), including 10 rectovaginal fistulas (2.3%), 1 intraperitoneal leak (0.2%), and 1 rectoureteral fistula (0.2%); no rectovesical fistulas occurred.

### 2.14. Details on the Complexity of Surgery

The number of operated organs and organs with an open lumen reflects the surgical complexity of the cohort. The number of operated organs is summarized in Table 7, and the number of additionally opened organs is presented in Table 8.

**Figure 1:**
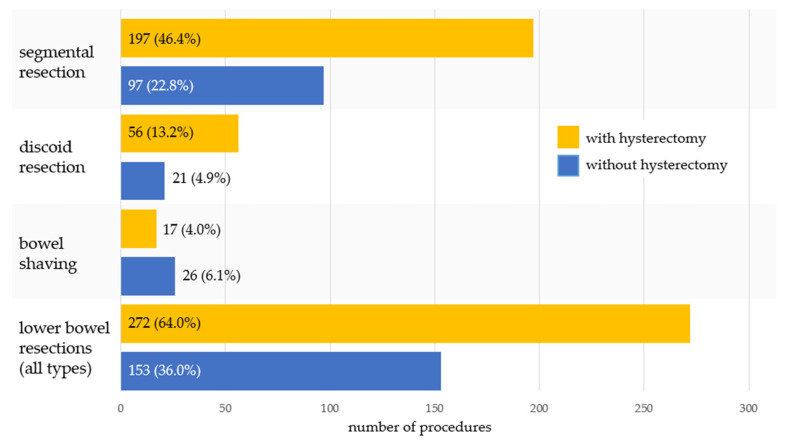
Frequency of lower bowel resection with and without hysterectomy.

Among lower bowel resections, procedures combined with hysterectomy predominated. This pattern was observed for both segmental and discoid resections. In contrast, bowel shaving was more often performed in procedures without hysterectomy. Details are presented in Figure 1.

**Figure 2:**
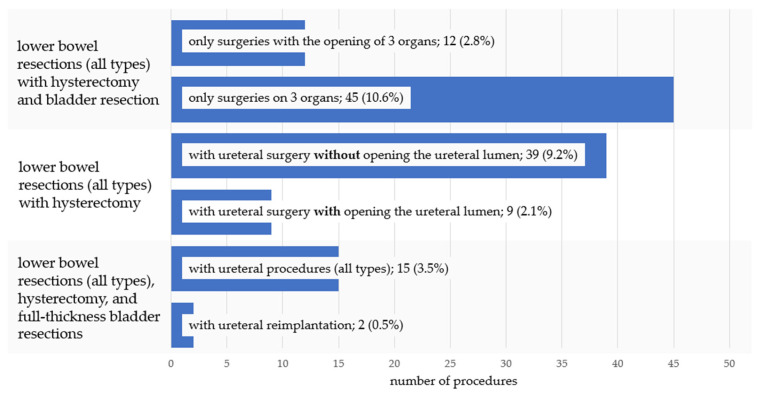
Frequency of lower bowel resection involving the urinary tract.

Lower bowel resections combined with hysterectomy were frequently associated with additional urinary tract procedures, reflecting the complexity of multiorgan surgery. Concomitant bladder and/or ureteral surgery was common, and a subset of cases required full-thickness bladder resection or ureteral reimplantation, indicating procedures with opening of the urinary tract lumen. The most extensive operations involved combined bowel, uterine, and urinary tract surgery. Detailed distributions are presented in Figure 2.

The surgical procedures performed in this cohort demonstrate a high degree of complexity, frequently involving multiorgan resections. Lower bowel resections were often combined with additional intestinal or organ procedures, including:

- Ileocecal resection (*n* = 27; 6.4%), representing 2 separate bowel resections;
- Ileocecal resection and small-bowel resection (*n* = 27; 6.4%), representing three separate segmental resections;
- Ileocecal resection and hysterectomy (*n* = 18; 4.2%), representing 2 separate bowel resections and additional organ (uterus).

The most extensive interventions included combined lower bowel resection with upper bowel resections, with operations on the urinary tract. In such cases, lower bowel resections were combined with:

- Ileocecal resection, upper bowel resection, hysterectomy, and bladder resection (*n* = 6; 1.4%), representing surgery on five organs, of which at least three had opened lumen;
- Ileocecal resection, upper bowel resection, full-thickness bladder resection, ureteral reimplantation (*n* = 6; 1.4%), representing surgery on five organs, of which at least four had opened lumen;
- Renal resection (*n* = 2; 0.5%).

The anastomotic level was categorized into four groups the distance between the colorectal anastomosis and the anal verge: high (≥8 cm), low (from 6 to <8 cm), very low (from 5 to <6 cm), and ultra-low (<5 cm). High anastomoses predominated across all resection types. A summary of the distribution by resection type is provided in Table 9.

Surgical complexity is reflected by the number of organs with an opened lumen during surgery. Table 10 shows the number of operated organs in the overall cohort and stratified by segmental and discoid resections, as well as by anastomotic height.

Rectovesical fistulae predominantly occurred in cases with lower resections. Table 11 shows the number of patients with two to five opened organs in the cohort, with the opening of the bowel lumen accounting for the anastomotic height in cases where a fistula occurred.

## 3. Results

Compared with patients without rectovaginal fistula, those who developed rectovesical fistula more frequently underwent segmental resection, had a lower anastomotic height, longer operative time, *Clostridioides difficile* infection, and involvement of the levator ani muscle. The occurrence of anastomotic leakage was not related to the method of anvil placement, whether performed via the vaginal, rectal, or sleeve technique. Risk factors for fistula occurrence are presented in Table 12.

The incidence of rectovaginal fistula was assessed across shaving, discoid, and segmental procedures, and further stratified by anastomotic height and anastomotic configuration. Fistulas occurred exclusively after segmental resections. The highest proportion was observed in the very-low-anastomosis subgroup (from 5 to <6 cm), which accounted for 40% of all fistulas within the very low segmental resections. No fistulas were observed after ultra-low anastomoses (<5 cm); however, all such cases were protected with a diverting stoma. Rectovaginal fistulas occurred in 6 patients undergoing concomitant hysterectomy, corresponding to an incidence of 3.0%. No fistulas were observed after shaving or discoid resection combined with hysterectomy, and within the segmental group, fistulas were most frequently associated with low and very low anastomoses. Details are presented in Table 13.

Concomitant vaginal lesion resection was associated with a higher incidence of rectovaginal fistula in the very low anastomosis subgroup. Overall, the fistula rate was higher in the vaginal lumen opening group than in the non-opening group (16.7% vs. 2.7%). The risk was greatest when the vaginal lumen was opened, but it also increased when the vaginal wall was thinned even without formal opening. Details are shown in Table 14.

Among patients undergoing discoid and segmental resections (*n* = 378), protective stomas were created predominantly in the segmental group (23 cases; 92%), while only 2 stomas (8%) were formed after discoid resection. Stoma formation was more frequent in lower anastomoses, particularly in the very low and ultra-low subgroups. All rectovaginal fistulas occurred exclusively after segmental resections, mainly in low and very low anastomoses, whereas no fistulas were observed in the ultra-low subgroup, likely due to protective stoma formation in all three cases (Table 15).

**Figure 3:**
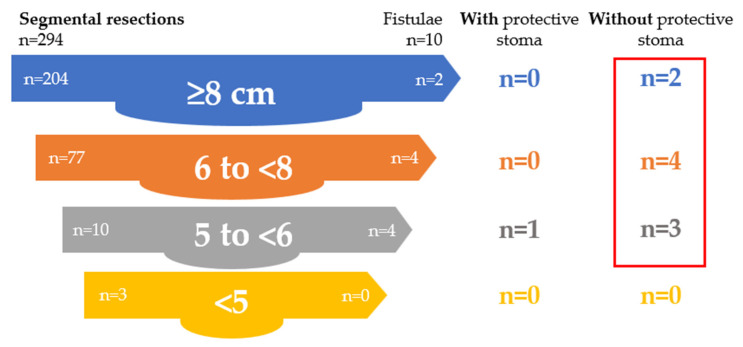
Role of protective stoma in preventing rectovaginal fistula after segmental resection.

In the segmental resection group (*n* = 294), rectovaginal fistulas occurred predominantly in patients without a protective stoma (90%) and were mainly associated with low and very low anastomoses. Protective stomas were associated with the absence of fistula formation in patients with high and low anastomoses, whereas a residual occurrence was observed in very low anastomoses, when the infiltration of levator ani was an additional risk factor. Details are presented in Table 16 and Figure 3.

**Figure 4:**
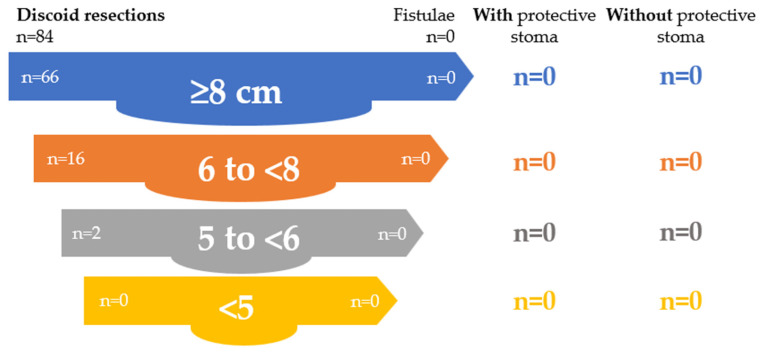
Role of protective stoma in preventing rectovaginal fistula after discoid resection.

In the discoid resection group, protective stomas were used in 2 patients (2.4%), both with low anastomoses. No rectovaginal fistulas occurred, and protective stoma formation was associated with the absence of fistulas in low anastomoses. Details are presented in Table 17 and Figure 4.

**Figure 5:**
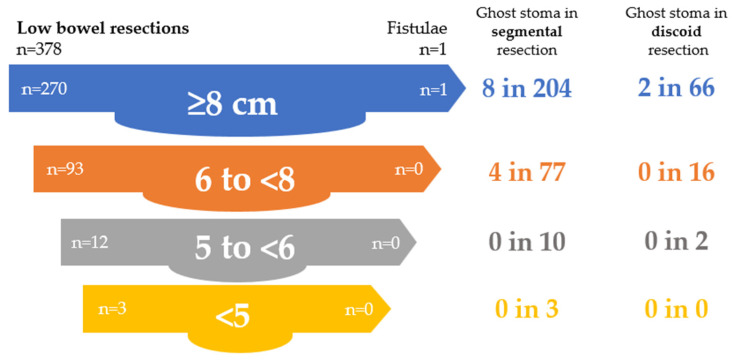
Role of the Ghost stoma in preventing rectovaginal fistula.

Ghost stomas were created in 14 cases (3.7%), predominantly in patients undergoing segmental resection. In high anastomoses, their use was similar between segmental and discoid procedures, whereas in low anastomoses, they were used only after segmental resections. Details are presented in Table 18 and Figure 5.

To explore whether a “safe” anastomotic height exists when deciding on concomitant hysterectomy, we evaluated the anastomotic level above which no rectovaginal fistulas were observed in patients with and without hysterectomy. In the hysterectomy group, no fistulas occurred when the anastomotic height was above 11 cm, whereas in the uterus-preserving group, no fistulas were observed above 10 cm. Overall, both thresholds were similarly high; however, in the uterus-preserving group, the “safe” anastomotic height was numerically 1 cm lower.

### Learning Curve

**Figure 6:**
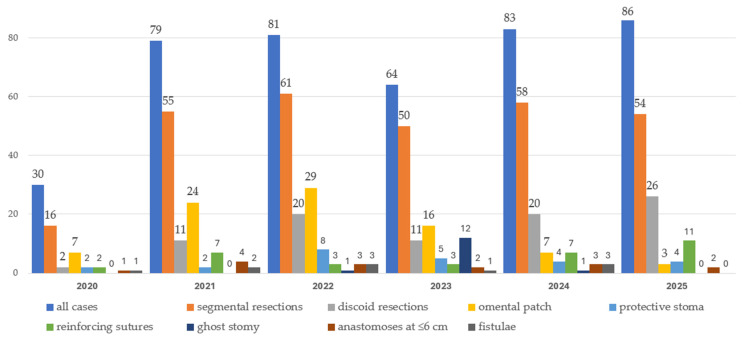
Comparison of resection types and protective measures over the study period from 2020 to 2025.

**Figure 7:**
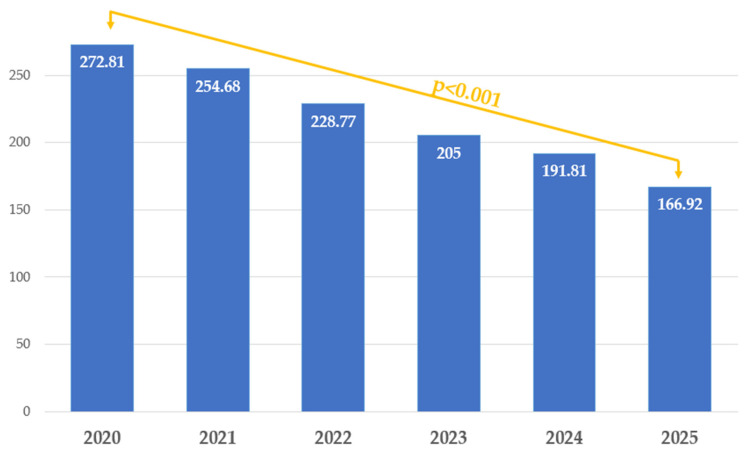
Comparison of operative time over the study period from 2020 to 2025.

Surgery duration decreased significantly over time (*p* < 0.0001), consistent with an improving learning curve. Over the study period, omental flap use declined markedly (*p* for trend *p* < 0.0001), whereas the use of reinforcing sutures, protective stomas, and Ghost stomas did not show a significant temporal trend. The use of Ghost stomas peaked in 2023. Changes in operative time and use of protective measures over the study period from 2020 to 2025 are depicted in Table 19 and in Figure 6 and Figure 7.

## 4. Discussion

The most serious mechanical complication of bowel anastomoses, carrying the highest risk of mortality, is anastomotic leakage. In the present study, 425 patients undergoing bowel resection for deep endometriosis were analyzed, and 421 of them underwent resection at the rectosigmoid level. All procedures were performed laparoscopically, and in all cases, indocyanine green fluorescence imaging was used to assess anastomotic perfusion and integrity.

In the entire cohort, intraperitoneal leakage occurred in only one patient (1/425; 0.2%). This case was associated with the incorrect intramural placement of a linear stapler during a side-to-side anastomosis, which necessitated re-resection and conversion to an end-to-end anastomosis. Subsequently, a perianastomotic microabscess developed, leading to anastomotic dehiscence. After modification of the linear stapler placement technique to eliminate the risk of intramural placement, no further such complications were observed. Needless to say, among all technically correct side-to-side anastomoses (*n* = 61) and circular stapler anastomoses (*n* = 294), no perioperative or postoperative anastomotic leakage occurred (Table 1 and Table 4). This is notable because anastomotic leakage is considered a major life-threatening complication, reported in approximately 1–2% of segmental resections according to Richards et al. [15], and as high as 3–6% in the study by Abo et al. [16]. In our cohort, when staplers were correctly inserted, and procedures were performed with high-quality devices by experienced surgeons, the occurrence of anastomotic leakage, understood as an intraperitoneal leak, was completely eliminated; only rectovaginal fistulas were detected. The observed rate of leakage was 0.2%, and the single event was attributable to a technical error, as explained above.

Another rare type of leakage was rectoureteral fistula, which occurred in one patient (1/425; 0.2%) who required concomitant ureteral dissection and segmental bowel resection. This complication developed during the period when indocyanine green fluorescence imaging was not yet used by the team to assess ureteral perfusion and when prophylactic ureteral stenting was not routinely applied. Although the impact of these measures on risk reduction cannot be definitively established, no further rectoureteral fistulas were observed after introducing this preventive approach in our practice [17].

Among all types of anastomotic leakages, rectovaginal fistula was the most common complication, occurring in 10 patients (10/425; 2.3%). All fistulas were observed in patients who underwent **segmental bowel resection**, and none occurred after discoid resection or bowel shaving. In contrast, Donnez and Roman [18] reported fistulas across all three bowel resection techniques in a large meta-analysis, with rates of 3.9% for segmental resection, 3.6% for discoid excision, and 1.3% for shaving procedures. In our cohort, the more conservative approaches—discoid resection and shaving—were not associated with fistula formation. Similarly, in a multicenter observational study, Hudelist et al. [19] reported fistula formation across all three types of bowel resection, with rates of 1.6% after segmental resection, 0.5% after discoid resection, and 0.9% after shaving. However, in the same cohort, the overall rate of anastomotic leakage was high: 3.6% for segmental resection, 1.4% for discoid resection, and 0.6% for shaving, resulting in a combined dehiscence rate of 8.6% across all procedures. The authors concluded that complication rates vary widely in low-volume centers. Although major complications tend to decrease as surgical volume increases, this effect cannot be generalized to all institutions or clinical settings. Similar fistula rates for discoid excision and segmental resection were also reported by Roman et al. [20]. In a systematic review and highlighted that multiple factors may influence these outcomes, including bowel preparation protocols, surgical technique, and, critically, surgeon experience [7]. According to meta-analysis, Bendifallah et al. [21], an annual threshold of approximately 20 colorectal endometriosis cases per center and 7–13 procedures per surgeon was associated with a significant reduction in grade III–IV complications. In our setting, the operating surgeon performed approximately 80–90 such surgeries per year, well above the proposed optimal threshold. Furthermore, performing only 20 cases per year does not provide sufficient surgical volume to reduce the risk of major complications in bowel surgery meaningfully.

Surprisingly, segmental bowel resection ranked only as the third strongest risk factor for rectovaginal fistula, whereas the presence of **levator ani muscle infiltration** emerged as the primary predictor (*p* < 0.0001). This was also the key factor in the only patient with a very low bowel anastomosis who developed a rectovaginal fistula despite having a protective stoma, performed because of a very low anastomosis combined with full-thickness vaginal excision. In all other cases, protective stomas prevented fistula development, including among patients with ultra-low anastomoses (<5 cm).

It should be noted that **anastomotic height** proved to be a stronger risk factor for fistula formation than the type of bowel resection. Similar observations were reported by Vigueras Smith et al. [7], who noted that anastomotic leakage can occur after any surgical modality, but is most frequently associated with low and ultra-low segmental resections.

It is important to reiterate that in our study, rectovaginal fistulas occurred exclusively in the segmental resection group. The highest incidence was observed in the very low anastomosis subgroup (from 5 to <6 cm), where 40% (4/10) of patients developed a fistula. One of these patients had a protective stoma; however, she also presented with the strongest identified risk factor: levator ani muscle infiltration. In contrast, no fistulas occurred in the ultra-low subgroup (<5 cm), despite concomitant vaginal opening and segmental resection, likely because all patients in this category were managed with a protective stoma. In one patient, a fistula developed despite an anastomotic height greater than 8 cm; however, this case involved extensive multiorgan resection, diabetes, and a BMI above 35. Several studies have shown that a BMI ≥ 35 kg/m^2^ is independently associated with an increased risk of anastomotic leakage [22,23].

Many authors have emphasized the importance of considering a protective stoma in cases of very low anastomoses (<5 cm) [24]. Malzoni et al. [25] reported that rectovaginal fistulas developed in 2.4% of patients in his cohort, occurring exclusively in those with ultra-low anastomoses and concomitant vaginal resection who did not receive a temporary stoma. In our study, 24 of 25 patients with a protective stoma did not develop a rectovaginal fistula; the single case occurred in a patient with levator ani infiltration described earlier, even among those with low (*n* = 8), very low (*n* = 6), or ultra-low (*n* = 3) anastomoses. What is more, in the discoid resection group, protective stomas also prevented fistula formation in patients with low or very low anastomoses, including those with levator ani muscle infiltration. These findings suggest that while protective stomas do not eliminate the risk of rectovaginal fistula entirely, they substantially reduce its occurrence. On the contrary, Roman et al. [20] reported different results, observing high fistula rates among patients undergoing concomitant vaginal surgery regardless of stoma use, with rates of 11.4% in patients with a stoma and 9.8% in those without a protective stoma. Nonetheless, randomized controlled trials and meta-analyses have shown that the use of a protective stoma in low colorectal anastomosis can substantially reduce morbidity and the clinical consequences of leakage, with reported reductions in symptomatic anastomotic leakage of up to 68% [26]. A meta-analysis from the Cochrane Library showed that the use of a protective stoma was significantly associated with a lower risk of anastomotic leakage (RR 0.33; 95% CI 0.21–0.53) and a reduced need for reoperation (RR 0.23; 95% CI 0.12–0.42) [27].

We also examined the association between concomitant vaginal or uterine procedures and fistula formation. Fistulas occurred almost exclusively (9/10) in patients who, in addition to segmental bowel resection, underwent **hysterectomy with vaginal shaving** (6/10), **vaginal endometriotic lesion excision** with lumen opening (2/10), or vaginal lesion excision without opening of its lumen (1/10). Although these factors did not reach statistical significance, the trends were notable for hysterectomy with vaginal lesion resection (*p* = 0.051) and vaginal lesion resection with lumen opening without hysterectomy (*p* = 0.068). Overall, every fistula in our cohort occurred in the subgroup undergoing segmental bowel resection combined with either vaginal excision or hysterectomy. In the study by Abo et al., rectovaginal fistula occurred in 3.8% of 364 bowel resections, with no significant differences between resection types; notably, 50% of these cases involved concomitant vaginal resection [16].

Operative time emerged as a particularly strong risk factor. It was unexpectedly comparable to anastomotic height and exceeded the impact of segmental resection (*p* = 0.001). A clear difference was observed: no fistulas occurred in patients whose procedures lasted an average of 211 min. Similar findings were reported by Huh et al. [28], who noted that the risk of anastomotic leakage increases markedly when operative time exceeds 220–300 min.

Interestingly, the **number of operated organs** was not associated with the occurrence of fistulas. Among patients who developed a fistula and underwent surgery on upper segments of the large bowel, procedures more often involved the colon (*p* = 0.523; *n* = 30) and appendix (*p* = 0.087; *n* = 47) than the small intestine (*p* = 1.0; *n* = 39). Urinary tract procedures were also not associated with the increased occurrence of fistulas. This included bladder surgery (*p* = 0.69; *n* = 77), ureteral reimplantation (*p* = 1.0; *n* = 14), and dissection of the ureters requiring DJ stent placement (*p* = 0.97; *n* = 82). However, when urinary leakage occurred (*p* = 0.09, *n* = 4), this factor, although not statistically significant, began to show a potential association with fistula formation.

In multiorgan surgeries, the number of operated organs did not play a role (*p* = 0.1507), unless their **lumens were opened** during surgery (*p* = 0.062). Hysterectomy with other procedures was not associated with fistula formation (*p* = 0.79), unless accompanied by vaginal infiltration requiring vaginal shaving (*p* = 0.051). In contrast, vaginal opening without hysterectomy showed a tendency toward higher fistula rates, although this did not reach statistical significance in our cohort, likely due to limited sample size (*p* = 0.068). The weak association between fistulas and concomitant hysterectomy with bowel resection may be explained by the routine use of multiple preventive measures in our study, including **fibrin glue** (*p* = 1), **omental flap** (*p* = 0.43), and **reinforcing sutures** (*p* = 0.56). Although none of these techniques, when analyzed individually, played any significant role, their combined application appears to have played a meaningful protective role. This may explain why our observations differ from previously published studies, in which such preventive methods were not used [7,29,30].

The **length of the resected bowel segment**, as well as the length of the infiltrating lesion, showed no association with an increased risk of fistula formation.

**Ghost stomas** did not prove a beneficial technique in the subgroup of patients in whom they were used. Among all 14 patients who received a Ghost stoma (14/294 in the discoid + segmental group), none required conversion to a protective stoma during the early postoperative period. However, one patient developed a rectovaginal fistula on postoperative day 30, after the Ghost stoma had been removed on day 7; this patient had insulin resistance, a BMI of 35, and a postoperative *Clostridioides difficile* infection. Notably, *C. difficile* infection was significantly associated with fistula formation (*p* = 0.025), emerging as a stronger predictor of this complication than the type of bowel resection. To the best of our knowledge, this is the first report describing such an association.

Surprisingly, the use of a pedicled **omental flap**, **reinforcing sutures**, or **fibrin glue** did not, when assessed individually, reduce the incidence of fistulas. However, when these techniques were applied in combination and incorporated into the team’s progressively refined preventive strategy, they contributed to a substantial decrease in fistula rates. As a result, in the last year of observation, no perioperative rectovaginal fistulas occurred. As shown in Figure 6 and Table 19, despite similar proportions of total bowel resections and protective stomas, and only minor differences in the ratio of segmental to discoid resections, the slightly higher use of reinforcing sutures in 2025 corresponded with the complete absence of fistulas in that year.

It appears that the strongest risk factor for fistula formation is segmental resection with a very low anastomosis and concomitant vaginal surgery, particularly when the vaginal lumen is opened. When this situation is further complicated by lateral infiltration of the levator ani muscle, the risk becomes critical. In such cases, discoid resection, which may limit the extent of vaginal surgery, can reduce the likelihood of fistula formation. In critical situations with very low anastomoses accompanied by levator ani involvement, the creation of a protective stoma serves as an important measure to lower fistula risk. Operative time also influences the likelihood of anastomotic failure, and efficiently performed procedures significantly decrease this risk. In multiorgan disease requiring simultaneous surgery on several organs, the extent of organ involvement alone does not increase fistula risk, provided that low anastomoses are not present.

A particularly insightful aspect of this study is the demonstration of how preventive strategies evolved over time. The proportion of less radical procedures, such as discoid resections, increased relative to segmental resections. Another indicator of the team’s growing proficiency was the progressive reduction in operative time, which decreased significantly over the years. The average operative time for multiorgan resections in 2025 in our institution, which was 166.92 min, and it was primarily bowel segmental resection, demonstrates a level of surgical proficiency comparable to leading international referral centers. For example, a highly experienced Brazilian multidisciplinary gynecologic–surgical team reported mean operative times of 166 min for shaving (*n* = 31) and discoid resection (*n* = 71), while 188 min for segmental resection (*n* = 71) [13]. Data from the TrEnd trial showed a median operative time of 160 min for laparoscopic segmental sigmoid and rectal resections performed for bowel endometriosis [25]. Although our median operative time was comparable, this is particularly meaningful considering that most of our procedures involved segmental bowel resection combined with additional surgery on the uterus, vagina, upper bowel, or urinary tract, making the overall surgical complexity substantially higher.

This improvement undoubtedly reflects increasing surgical experience, but also a shift toward lower procedural complexity. In the first two years, operations were more complex compared with the subsequent four years. We had three cases involving extremely extensive procedures, including surgery on 4–5 organs, such as nephrectomy performed during the same operation or creation of an ileal neobladder in addition to bowel resection and hysterectomy, really major procedures. Although we routinely manage complex cases, operations involving 2–3 organs are far more common than those requiring surgery on 4–5 organs. This was largely because, during its initial period of activity, OCO was the only public institution in Poland performing such advanced deep endometriosis surgery. As additional centers gradually began offering similar procedures, the number of the most neglected and complex cases referred to OCO diminished. Consequently, the case mix shifted toward less complicated procedures, contributing further to shorter operative times and the refinement of preventive strategies.

In 2023, Ghost stomas were introduced; however, they did not influence perioperative management and ultimately did not prove useful in practice. In 2024, the team critically limited bowel resection for lesions located less than 5 cm from the anal verge and increased the use of discoid resections for low-lying lesions, particularly when vaginal infiltration was present. The year 2025 reflects a well-balanced surgical practice tailored to patient needs. It was characterized by an appropriate proportion of discoid and segmental resections, judicious use of preventive measures such as omental flaps, reinforcing sutures, and protective stomas, and, notably, it was a year without a single postoperative fistula.

Several limitations must be acknowledged. The number of rectovaginal fistula cases was small (*n* = 10; 2.3%), which limits the statistical power for identifying risk factors. Similarly, the shaving group was too small to allow a reliable assessment of outcomes for this technique. The study primarily compares segmental and discoid resections, which may not capture the full spectrum of surgical approaches used in deep endometriosis. Additionally, although the single-center design ensured a standardized surgical approach, it may limit the generalizability of the findings, and the lack of long-term follow-up for the entire cohort restricted the evaluation of long-term outcomes.

## 5. Recommendations for Gynecological Surgeons

Based on our clinical experience and the findings of this study, we developed a set of recommendations aimed at reducing the risk of complications associated with low bowel resections for deep endometriosis.

1. The risk of anastomotic leakage is minimal when low rectal and sigmoid anastomoses are performed correctly and with high-quality stapling devices.
2. The strongest risk factors for rectovaginal fistula are low anastomoses performed during segmental resection, particularly when accompanied by lateral infiltration of the levator ani muscle and concomitant hysterectomy or vaginal opening.
3. For low and very low anastomoses in cases where vaginal opening is likely, discoid resection should be considered the preferred surgical technique.
4. Protective stomas significantly reduce, although do not completely eliminate, the risk of fistula formation in very low (5–6 cm from the anal verge) or ultra low anastomoses. (below 5 cm).
5. In patients with the co-occurrence of levator ani muscle infiltration undergoing concomitant surgery on the bowel and the vagina with low anastomosis location, the risk of fistula formation is high and may not be fully mitigated by a protective stoma. In such cases, performing the vaginal and bowel procedures in separate stages is recommended.
6. Factors such as the number of previous surgeries, overall surgical complexity (number of operated organs, the number of opened organs) do not affect the risk of anastomotic failure.
7. Despite the high complexity of procedures, the use of preventive techniques resulted in a low overall rate of rectovaginal fistulas (10/421; 2.3%) in patients undergoing lower bowel resection.
8. Operative time is an important risk factor for postoperative complications, including rectovaginal fistula.

## 6. Conclusions

Rectovaginal fistulas occurred exclusively after segmental bowel resection, particularly when the anastomosis was very low (5–6 cm from the anal verge) and accompanied by infiltration of the levator ani muscle as well as simultaneous vaginal or uterine procedures. In such high-risk situations, discoid resection represents a safer surgical alternative when technically feasible. Despite the overall complexity of the operations performed, the consistent use of preventive strategies kept the rate of rectovaginal fistulas low, and protective stomas markedly reduced the occurrence of such complications in patients with very low anastomoses.

## Figures and Tables

**Table 1:** Sizes circular stapler used (*n* = 363).

	33 mm Stapler	31 mm Stapler	28 mm Stapler
Number (%) of cases	344 (94.8%)	18 (4.9)	1 (0.3%)

**Table 2:** Distribution of all segmental resections of the bowel (*n* = 438).

	Resected Segment	Number (%)
Resected segments of the lower bowel	Rectum	52 (11.9%)
	Sigmoid colon	83 (18.9%)
	Rectum and sigmoid colon	159 (36.3%)
Resected segments of the upper bowel (proximal to the sigmoid colon) accompanying lower bowel resections	Transverse colon and lower bowel resection *	1 (0.2%)
	Cecum and lower bowel resection *	30 (6.9%)
	Appendix with concomitant upper bowel resection ^†^ and lower bowel resection *	47 (10.7%)
	Appendix alone and lower bowel resection *	16 (3.7%)
	Small intestine with concomitant upper bowel resection ^†^ and lower bowel resection *	38 (8.7%)
	Small intestine and lower bowel resection *	8 (1.8%)
Other	Upper bowel resection ^†^ without lower bowel resection *	4 (0.9%)

* Resection of lower bowel segment denotes resection of the rectum, sigmoid colon, or rectum and sigmoid colon; ^†^ Resection of the upper bowel segment denotes resection of the cecum, appendix, and terminal ileum together.

**Table 3:** Types of procedures on the lower bowel (total: rectum/sigmoid colon/rectum and sigmoid colon; *n* = 421).

Type of Resection	Number (%)
Bowel shaving	43 (10.2%)
Discoid resection only	68 (16.2%)
Discoid resection and shaving	16 (3.8%)
Discoid resection and segmental resection	4 (9.5%)
All discoid resections *	84 (20.0%)
All segmental resections	294 (69.8%)

* From the discoid resection group, surgeries with segmental resections performed at the same operation were excluded.

**Table 4:** Types of bowel anastomoses in lower segmental resections (*n* = 294).

Type of Anastomosis	Side-to-Side (ss)	End-to-End (ee)	Side-to-End (se)	End-to-Side (es)
Number (%)	15 (5.1%)	263 (89.4%)	9 (3.1%)	7 (2.4%)

**Table 5:** Methods of anvil placement (*n* = 294).

Type of Anvil Placement	Fishing Per Vaginam (FP)	Per Rectum (F)	Per Surgisleeve (SS)	Intra-Abdominal Placement of a Linear Stapler in Sigmoid—Sigmoid Anastomoses
Number (%)	85 (28.9%)	80 (27.2%)	114 (38.8%)	15 (5.1%)

**Table 6:** Types of ureteral procedures.

Types of Procedures	1 DJ Only	2 DJ Only	Reimplantations (+DJ Always)	Shaving	Resection/Suturing	Lower Bowel Resections with Ureteral Procedure
Number	58	37	14	43	12	164
Relative to the entire group of patients undergoing ureteral procedures (*n* = 164)	35.4%	22.6%	8.5%	26.2%	7.3%	100%
Percentage relative to all operated patients (*n* = 421)	13.8%	8.9%	3.3%	10.2%	2.9%	38.9%

**Table 7:** Number of additional organs operated in all groups of patients with low resection of bowel: segmental, discoidal, and shaving resection of the bowel (*n* = 421) *.

Number of Additional Organs Operated	Sigmoid/Rectum Only	1	2	3	4
Number (%)	82 (19.5%)	228 (54.2%)	78 (18.5%)	29 (6.9%)	4 (0.9%)

* Bowel shaving procedures were counted as shaving of a single organ. Cases on upper bowel were excluded.

**Table 8:** Number of opened organs during surgery in the group of segmental and discoidal resection (*n* = 378) *.

Number of Operated Organs	1	2	3	4	5	6
Number (%)	82 (21.8%)	223 (59%)	56 (14.9%)	15 (3.7%)	1 (0.3%)	1 (0.3%)

* Shaving procedures were excluded because shaving is not associated with organ opening.

**Table 9:** Anastomotic height distribution by resection type.

Anastomotic Height	All Resections with Opening of the Bowel Lumen	Segmental Resection	Discoid Resection
High (≥8 cm)	270 (71.4%)	204 (69.4%)	66 (78.6%)
Low (from 6 to <8 cm)	93 (24.6%)	77 (26.2%)	16 (19.0%)
Very low (from 5 to <6 cm)	12 (3.2%)	10 (3.4%)	2 (2.4%)
Ultra-low (<5 cm)	3 (0.8%)	3 (1.0%)	0
Total	378	294	84

**Table 10:** Details on the number of organs opened during surgery stratified by surgery type and by anastomotic height.

Anastomotic Height	One	Two	Three	Four	Five	Six
All (*n* = 384)	82 (21.4)	228 (53.6%)	57 (13.4%)	15 (3.5%)	1 (0.2%)	1 (0.2%)
High (≥8 cm)	60 (73.2%)	160 (71.1%)	39 (70.9%)	9 (60.0%)	1 (100%)	0
Low (from 6 to <8 cm)	17 (20.7%)	56 (25.1)	13 (23.6%)	6 (40%)	0	1 (100%)
Very low (from 5 to <6 cm)	4 (4.9%)	6 (2.7%)	2 (3.6%)	0	0	0
Ultra-low (<5 cm)	1 (1.2%)	1 (0.4%)	1 (1.8%)	0	0	0
All segmental resection (*n* = 294)	64 (21.8%)	173 (58.8%)	41 (13.9%)	14 (4.8%)	1 (0.3%)	1 (0.3%)
High (≥8 cm)	60 (73.2%)	160 (71.1%)	39 (70.9%)	9 (60.0%)	1 (100%)	0
Low (from 6 to <8 cm)	17 (20.7%)	56 (25.1)	13 (23.6%)	6 (40%)	0	1 (100%)
Very low (from 5 to <6 cm)	4 (4.9%)	6 (2.7%)	2 (3.6%)	0	0	0
Ultra-low (<5 cm)	1 (1.2%)	1 (0.4%)	1 (1.8%)	0	0	0
All discoid resection (*n* = 84)	18 (21.4%)	50 (59.5%)	15 (17.9%)	1 (1.2%)	0	0
High (≥8 cm)	16 (88.9%)	37 (74%)	13 (86.7%)	0	0	0
Low (from 6 to <8 cm)	2 (11.1%)	11 (22.0%)	2 (13.3%)	1 (100%)	0	0
Very low (from 5 to <6 cm)	0	2 (4.0%)	0	0	0	0
Ultra-low (<5 cm)	0	0	0	0	0	0

**Table 11:** Details on the number of organs opened during surgery stratified by anastomotic height and the number of rectovesical fistulae in each subgroup.

Total (*n* = 378)	One (*n* = 82)	Two (*n* = 223)	Three (*n* = 55)	Four (*n* = 15)	Five (*n* = 1)	Six (*n* = 1)
High (≥8 cm)	60 (73.2%) 0	160 (71.1%) 1/160 (0.6%)	39 (70.9%) 1/39 (2.5%)	9 (60%) 0	1 (100%) 0	0
Low (from 6 to <8 cm)	17 (20.7%) 0	56 (25.1) 2/56 (3.5%)	13 (23.6%) 2/13 (15%)	6 (40%)	0	1 (100%) 0
Very low (from 5 to <6 cm)	4 (4.9%) 1/4 (25%)	6 (2.7%) 2/6 (33.3%)	2 (3.6%) 1/2 (50%)	0	0	0
Ultra-low (<5 cm)	1 (1.2%) 0	1 (0.4%) 0	1 (1.8%) 0	0	0	0

**Table 12:** Risk factors for fistula occurrence.

Risk Factor	Cases Without Rectovaginal Fistula	Cases with Rectovaginal Fistula	*p*-Value
Shaving alone	43 (100%)	0 (0%)	0.6080
Discoid resection alone	68 (100%)	0 (0%)	0.3759
Discoid and segmental resection	6 (100%)	0 (0%)	1
Discoid resection and shaving	16 (100%)	0 (0%)	0.5273
Segmental resection	284 (96.6%)	10 (3.4%)	**0.0355**
Stapler placement	bb, 15 (100%) f, 69 (95.8%) fp, 86 (96.6%) ss, 110 (96.5%)	0 (0%) 3 (4.2%) 3 (3.4%) 4 (3.5%) 114	0.8849
Anastomotic height	8.97 ± 2.21	6.65 ± 2.12	**0.0010**
Ileocecal anastomosis	29 (96.7%)	1 (3.3%)	0.523
Upper bowel resection	53 (94.6%)	3 (5.4%)	0.1326
Appendix alone	14 (87.5%)	2 (12.5%)	**0.0502**
Appendix with other procedures	44 (93.6%)	3 (6.4%)	0.0872
Small intestine alone	7 (100.0%)	0 (0%)	1
Small intestine with other procedures	38 (97.4%)	1 (2.6%)	1
Number of additionally operated organs	1.17 ± 0.89	1.50 ± 0.71	0.1507
Number of opened organs	1.87 ± 0.88	2.30 ± 0.67	0.0626
Hysterectomy with additional vaginal opening	266 (97.8%)	6 (2.2%)	0.7515
Vaginal infiltration with vaginal opening without hysterectomy	17 (89.5%)	2 (10.5%)	0.0688
Vaginal infiltration without hysterectomy and without vaginal opening	64 (98.5%)	1 (1.5%)	1
Hysterectomy with vaginal infiltration	91 (94.8%)	5 (5.2%)	**0.051**
Hysterectomy with other procedures	266 (97.8%)	6 (2.2%)	0.7900
DJ catheter	0, 281 (97.6%) 1, 82 (97.6%) 2, 52 (98.1%)	0, 7 (2.4%) 1, 2 (2.4%) 2, 1 (1.9%)	0.9714
Size of lower bowel infiltrates, cm	4.41 ± 2.66	5.05 ± 2.24	0.2652
Bladder	76 (98.7%)	1 (1.3%)	0.6977
Ureter	63 (95.5%)	3 (4.5%)	0.2016
Hysterectomy with segmental or discoid resection	247 (97.6%)	6 (2.4%)	0.9756
Retransplantation	14 (100.0%)	0 (0.00%)	1
Duration of surgery	211.29 ± 84.38	309.00 ± 95.97	**0.0010**
Fibrin glue	366 (97.6%)	9 (2.4%)	1
Omental flap	83 (96.5%)	3 (3.5%)	0.4301
Reinforcing sutures	32 (97.0%)	1 (3.0%)	0.5584
Trachelectomy	4 (80.0%)	1 (20.0%)	0.1127
Urine Leak	3 (75.0%)	1 (25.0%)	0.0912
Other complications	20 (100.0%)	0 (0.0%)	1
Number of prior surgeries	1.64 ± 1.53	1.70 ± 1.49	0.8039
Length of lower bowel resection	7.85 ± 4.50	9.25 ± 5.25	0.3553
Clostridioides difficile	17 (4.1%)	3 (30.0%)	**0.0001**
Involvement of the levator ani muscle	15 (71.4%)	6 (28.6%)	**<0.0001**
BMI, kg/m^2^	23.5 ± 3.5	22.2 ± 3.2	0.1702
Age, year	37.7 ± 6.0	36.6 ± 5.5	0.5347

Bold denotes statistical significance.

**Table 13:** Occurrence of rectovaginal fistulae by surgical technique and anastomotic height in surgeries of different complexity.

Anastomotic Height, cm	Shaving	Anastomoses in Discoid Resections (No Anastomoses/No Patients)	Anastomoses in Segmental Resections (No Anastomoses/No Patients)
**Entire cohort: shaving (*n* = 43), discoid resections (*n* = 84), segmental resections (*n* = 294)**			
High (≥8 cm)	NA	0/66 (0%)	2/204 (0.98%)
Low (from 6 to <8 cm)	NA	0/16 (0%)	4/77 (5.2%)
Very low (from 5 to <6 cm)	NA	0/2 (0%)	4/10 (40%)
Ultra-low (<5 cm)	NA	0/0 (0%)	0/3 (0%)
Total	0	0/66 (0%)	10/294 (3.4%)
**With concomitant hysterectomy: shaving (*n* = 17), discoid resections (*n* = 56), segmental resections (*n* = 197)**			
High (≥8 cm)	NA	0/44 (0%)	1/140 (0.7%)
Low (from 6 to <8 cm)	NA	0/10 (0%)	4/50 (8.0%)
Very low (from 5 to <6 cm)	NA	0/2 (0%)	1/4 (25%)
Ultra-low (<5 cm)	NA	0/0 (0%)	0/2 (0%)
Total anastomoses	0	0/56 (0%)	6 (3.0%)

NA, not applicable, as anastomoses are created only in discoid and segmental resections.

**Table 14:** Occurrence of rectovaginal fistulae following segmental resections by type of vaginal surgery.

Anastomotic Height, cm	Vaginal Tumor Resection with Vaginal Lumen Opening and Segmental Bowel Resection (*n* = 12) (No Anastomoses/No Patients)	Vaginal Tumor Resection Without Vaginal Lumen Opening and Segmental Bowel Resection (*n* = 37) (No Anastomoses/No Patients)
High (≥8 cm)	0/4 (0%)	0/22 (0%)
Low (from 6 to <8 cm)	0/5 (0%)	0/12 (0%)
Very low (from 5 to <6 cm)	2/3 (66.7%)	1/2 (50%)
Ultra-low (<5 cm)	0/0 (0%)	0/1 (0%)
Total anastomoses	2/12 (16.7%)	1/37 (2.7%)

**Table 15:** Protective stoma use by surgical technique and anastomotic height, with rectovaginal fistula occurrence.

Anastomotic Height, cm	All Patients	All Protective Stomas	Segmental Resections/ Stomas	Discoid Resections/ Stomas	Rectovaginal Fistulas
High (≥8 cm)	270 (71.4%)	8 (32%)	204 (69.4%) 8/204 (3.9%)	66 (78.6%) 0/66 (0%)	2 *
Low (from 6 to <8 cm)	93 (24.6%)	8 (32%)	77 (26.2%) 8/77 (10.4%)	16 (19%) 0/16 (0%)	4 *
Very low (from 5 to <6 cm)	12 (3.2%)	6 (24%)	10 (3.4%) 4/10 (40%)	2 (2.4%) 2/2 (100%)	4 *
Ultra-low (<5 cm)	3 (0.8%)	3 (12%)	3 (1%) 3/3 (100%)	0/0 (0%) 0	0
Total	378	25	23/294 (7.8%) 23/25 (92%)	2/84 2/25 (8%)	10

* Rectovaginal fistula developed only following a segmental resection.

**Table 16:** Role of protective stoma in preventing rectovaginal fistula after segmental resection.

Anastomotic Height, cm	Segmental Resection (*n* = 294)	Fistula (*n* = 10)	Fistula with Protective Stoma	Fistula Without Protective Stoma
High (≥8 cm)	204 (69.4%)	2/204 (0.98%)	0 No cases of fistula	2/2 (100%) All without stoma developed fistula
Low (from 6 to <8 cm)	77 (26.2%)	4/77 (5.2%)	0 No cases of fistula	4/4 (100%) All without stoma developed fistula
Very low (from 5 to <6 cm)	10 (3.4%)	4/10 (40%)	1/4 (25%) 25% developed a fistula despite stoma protection	3/4 (75%) 75% without stoma developed fistula
Ultra-low (<5 cm)	3 (1%)	-	0 No cases of fistula	0 No cases of fistula

**Table 17:** Role of protective stoma in preventing rectovaginal fistula after discoid resection.

Anastomotic Height, cm	Discoid Resection (*n* = 84)	Protective Stoma (*n* = 2)	Fistula with Protective Stoma	Fistula Without Protective Stoma
High (≥8 cm)	66 (78.6%)	0/66 (0%)	0 No cases of fistula	0 No cases of fistula
Low (from 6 to <8 cm)	16 (19%)	0/16 (0%)	0 No cases of fistula	0 No cases of fistula
Very low (from 5 to <6 cm)	2 (2.4%)	2/2 (100%)	0 No cases of fistula	0 No cases of fistula
Ultra-low (<5 cm)	-	-	-	-

**Table 18:** Role of Ghost stoma in preventing rectovaginal fistula.

Anastomotic Height, cm	All Cases/ Ghost Stoma	Segmental Resection/ Ghost Stoma	Discoid Resection/ Ghost Stoma	Fistula
High (≥8 cm)	270 (71.4%) 10/270 (3.7%)	204 (69.4%) 8/204 (3.9%)	66 (78.6%) 2/66 (3%)	1 *
Low (from 6 to <8 cm)	93 (24.6%) 4/93 (4.3%)	77 (26.2%) 4/77 (5.2%)	16 (19%) 0/16 (0%)	0
Very low (from 5 to <6 cm)	12 (3.2%) 0/12 (0%)	10 (3.4%) 0/10	2 (2.4%) 0/2 (0%)	0
Ultra-low (<5 cm)	3 (0.8%) 0/3 (0%)	3 (1%) 0/3 (0%)	-	0
Total	378 14/378 (3.7%)	294 12/294 (4.1%)	84 2/84 (2.4%)	0

* Rectovaginal fistula developed only following a segmental resection.

**Table 19:** Duration of surgery and the use of protective measures in subsequent years.

	Surgery Duration, min	Reinforcing Sutures	Omental Flap	Protective Stoma	Ghost Stoma
2020 *n* = 32	272.81 ± 134.66 (a) (b) (c)	2 (6.2%)	7 (21.9%)	2 (6.2%)	0 (0%)
2021 *n* = 79	254.68 ± 81.52 (d) (e) (f)	7 (8.9%)	24 (30.4%)	2 (2.5%)	0 (0%)
2022 *n* = 81	228.77 ± 84.42 (g)	3 (3.7%)	29 (35.8%)	8 (9.9%)	1 (1.2%)
2023 *n* = 64	205.00 ± 58.67 (a) (d)	3 (4.7%)	16 (25.0%)	5 (7.8%)	12 (18.8%)
2024 *n* = 83	191.81 ± 73.02 (b) (e)	7 (8.4%)	7 (8.4%)	4 (4.8%)	1 (1.2%)
2025 *n* = 86	166.92 ± 61.67 (c) (f) (g)	11 (12.8%)	3 (3.5%)	4 (4.7%)	0 (0%)
*p*-value	<0.0001 *	0.3031 ^†^	<0.0001 ^†^	0.4434 ^†^	<0.0001 ^†^
*p* for trend	-	0.1683 ^†^	<0.0001 ^†^	0.8681 ^†^	0.6557 ^†^

* Kruskal–Wallis test; (a) groups with the same letter differ significantly in the post hoc test according to Conover; ^†^ Chi-squared test.

## Data Availability

Dataset available on request from the authors.

## Abbreviations

The following abbreviations are used in this manuscript:

BMI	Body mass index
ee	End-to-end anastomosis
es	End-to-side anastomosis
F	Fishing per rectum
FP	Fishing per vaginam
ICG	Indocyanine green
MRI	Magnetic resonance imaging
OCO	Opole Oncology Center
RVF	Rectovaginal fistula
se	Side-to-end anastomosis
ss	Side-to-side anastomosis
SS	Per Surgisleeve
TVUS	Transvaginal ultrasound
